# Association and Agreement between Reactive Strength Index and Reactive Strength Index-Modified Scores

**DOI:** 10.3390/sports9070097

**Published:** 2021-07-05

**Authors:** Talin Louder, Brennan J. Thompson, Eadric Bressel

**Affiliations:** 1 Department of Kinesiology and Health Science, Utah State University, Logan, UT 84322, USA; brennan.thompson@usu.edu (B.J.T.); eadric.bressel@usu.edu (E.B.); 2 Dennis G. Dolny Movement Research Clinic, Sorenson Legacy Foundation Center for Clinical Excellence, Utah State University, Logan, UT 84322, USA

**Keywords:** reactive strength index, reactive strength index-modified, plyometric, agility

## Abstract

Since the reactive strength index (RSI) and reactive strength index-modified (RSI-mod) share similar nomenclature, they are commonly referred as interchangeable measures of agility in the sports research literature. The RSI and RSI-mod are most commonly derived from the performance of depth jumping (DJ) and countermovement jumping (CMJ), respectively. Given that DJ and CMJ are plyometric movements that differ materially from biomechanical and neuromotor perspectives, it is likely that the RSI and RSI-mod measure distinct aspects of neuromuscular function. The purpose of this investigation was to evaluate the association and agreement between RSI and RSI-mod scores. A mixed-sex sample of NCAA division I basketball athletes (*n* = 21) and active young adults (*n* = 26) performed three trials of DJ from drop heights of 0.51, 0.66, and 0.81 m and three trials of countermovement jumping. Using 2-dimensional videography and force platform dynamometry, RSI and RSI-mod scores were estimated from DJ and CMJ trials, respectively. Linear regression revealed moderate associations between RSI and RSI-mod scores (*F* = 11.0–38.1; *R*^2^ = 0.20–0.47; *p* < 0.001–0.001). Bland–Altman plots revealed significant measurement bias (0.50–0.57) between RSI and RSI-mod scores. Bland–Altman limit of agreement intervals (1.27–1.51) were greater than the mean values for RSI (0.97–1.05) and RSI-mod (0.42) scores, suggesting poor agreement. Moreover, there were significant performance-dependent effects on measurement bias, wherein the difference between and the mean of RSI and RSI-mod scores were positively associated (*F* = 77.2–108.4; *R*^2^ = 0.63–0.71; *p* < 0.001). The results are evidence that the RSI and RSI-mod cannot be regarded as interchangeable measures of reactive strength.

## 1. Introduction

The stretch-shortening cycle (SSC) is a natural action involving the stretch, or eccentric lengthening, of an active skeletal muscle immediately prior to contraction [1]. The primary role of the SSC is to optimize mechanical loading of the muscle-tendon complex, which may lead to a metabolically efficient and forceful muscle contraction [2]. Neurophysiological mechanisms responsible for SSC enhancement of muscle performance are debated [2], yet may include storage and recapture of elastic potential energy [2,3,4,5,6], skeletal muscle pre-activation [3,4,5,6], increased active state [2], residual force enhancement [3,4,5,6], pre-synaptic facilitation of alpha motor neurons from supraspinal drive [7], and involuntary spinal reflex pathways [3,4,5]. Given the role of the SSC in optimizing muscle efficiency and force, it is essential to identify measures that effectively differentiate SSC utilization during functional movement tasks.

Reactive strength was first introduced by Warren Young [8] as a measure of lower-extremity SSC utilization in jumping. Specifically, Young [8] defined reactive strength as “The ability to utilize stretching of the muscle and then change quickly from an eccentric to a concentric contraction”. Jumping movements that involve high stretch-loads and ground contact times (GCTs) ≤ 250 ms are categorized as fast SSC, with jumping movements involving GCTs ≥ 251 ms categorized as slow SSC [9]. To measure slow SSC utilization, Young [8] proposed taking the difference between jump heights (JHs) achieved using the countermovement (CMJ) and squat jumping (SJ) techniques (Table 1). To measure fast SSC utilization, Young [8] established a metric known presently as the Reactive Strength Index (RSI).

The RSI is computed from jumping movements comprising a distinct ground contact, or impact, phase by taking a ratio of JH to GCT (Table 1). The RSI was introduced as a measure of depth jump (DJ) performance. DJ was introduced as the “shock method” of training by Yuri Verkhoshanksy [10] and has persisted as a jumping movement commonly included in plyometric training programs targeting SSC enhancement [11]. The DJ technique involves a maximal vertical jump performed immediately following landing impact from a self-initiated drop. DJ is considered a fast SSC movement, however, it is common for GCTs to exceed the 250 ms threshold when JH is emphasized through verbal instruction or when DJs are performed from a high drop [12]. For example, Struzik et al. [13] observed significantly longer *GCT*s when DJs were performed with verbal instruction to maximize JH (GCT = 0.33–0.43 s) versus instruction to both maximize JH and minimize GCT (GCT = 0.23–0.28 s). Further, Addie et al. [14] observed significantly longer GCTs in a sample of active young adults when DJs were performed from drop heights of 0.76 and 0.91 m versus drop heights ranging between 0.30 and 0.60 m.

The RSI was recently modified by Ebben and Petushek [15] for application as an assessment of slow SSC utilization in CMJ. The RSI-modified (RSI-mod) is computed by replacing GCT with time to take-off (TTT; Table 1), a temporal variable representing the duration between countermovement initiation and CMJ take-off.

The RSI and RSI-mod share similar nomenclature and both fit within Young’s [8] original definition of reactive strength, yet their discrimination of lower-extremity reactive strength, or SSC utilization, may depend on jumping technique [16,17,18]. For example, DJ and maximal repetitive jumping are the most common jumping techniques used to estimate the RSI. The 10/5 repeated jump test (10/5 RJT) is a maximal repetitive jumping technique wherein 10 bilateral jumps are performed immediately after an initial CMJ [18]. Although DJ and the 10/5 RJT both comprise a distinct ground contact phase and involve maximal jumps for height subsequent to a landing impact, Stratford et al. [17] observed only a moderate linear association (*R*^2^ = 0.30) between RSI scores derived from DJ and the 10/5 RJT. While there is likely a multitude of factors that distinguish the 10/5 RJT from DJ, Stratford et al. [17] comment on conceivable differences in the feedforward neuromotor control of landing impact, noting that the DJ technique allows for a motor response to be planned in advance of the self-initiated drop.

In contrast with CMJ, DJ tends to elicit shorter GCTs [16], greater ground reaction force (GRF) magnitudes and greater rates of GRF development (RFD; [19]). Additionally, DJ does not involve a prolonged unweighting phase and requires complex feedforward and feedback neuromotor control of a drop and landing phase whereas CMJ is performed entirely with the feet in contact with the ground [20,21,22,23]. Notably, the differences between DJ and CMJ provide a basis for questioning whether the RSI and RSI-mod are compatible measures of reactive strength. Recently, McMahon et al. [16] observed a moderate linear association (*R*^2^ = 0.22) between RSI-mod (CMJ) and RSI (0.30 m DJ) scores in a sample of professional male rugby athletes. The 22% shared variance reported by McMahon [16] is evidence that the RSI and RSI-mod are somewhat distinct, yet it is also important to mention that results from linear regression do not necessarily reflect the extent of agreement between measures [24,25,26].

**Figure 1:**
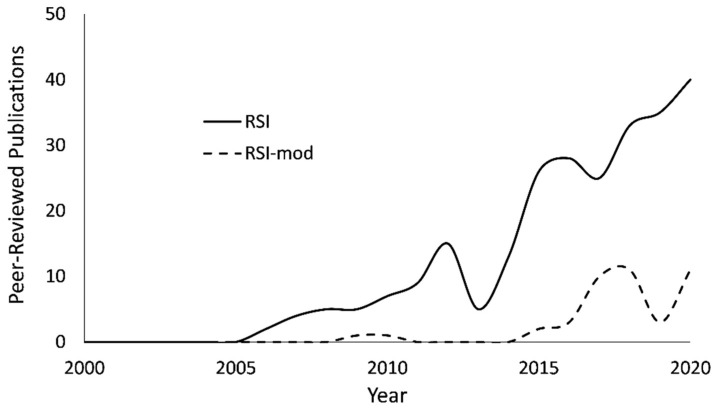
Yearly (2000–2020) count of peer-reviewed publications that include the reactive strength index (RSI) or reactive strength index-modified (RSI-mod) as dependent measures. Results are from an informal Google Scholar search that was performed using the search term “reactive strength index”. Manuscripts were counted from the first 400 entries returned for each calendar year.

To address the limitations of linear regression, Bland and Altman [24] proposed an alternative analysis that provides meaningful discernment through informal interpretation of limits of agreement (95% CI) and measurement bias [25,26]. A comprehensive analysis of the association and agreement between RSI and RSI-mod scores is timely considering the recent and increasing interest of both measures in the literature (Figure 1). Further, with the RSI and RSI-mod having been applied across diverse populations, a representative analysis of measure compatibility may be comprised of a mixed-sex sample of participants with varied reactive strength ability as opposed to a homogenous sample. Therefore, the purpose of the present investigation was to evaluate the association and agreement between RSI and RSI-mod scores acquired from a mixed-sex sample of National Collegiate Athletic Association (NCAA) division I basketball athletes and active young adults. NCAA athletes and recreationally active young adults are among the most common populations studied in the RSI and RSI-mod literature. The decision to include a mixed-population sample was made to provide representation of the literature and to strengthen the analysis of association and agreement of RSI and RSI-mod scores by means of measuring a broad range of jumping ability. We hypothesized that there would be a significant linear association between RSI and RSI-mod scores, yet the totality of evidence would not support application of the RSI and RSI-mod as interchangeable measures of reactive strength.

## 2. Materials and Methods

### 2.1. Participants

Twenty-one NCAA Division I basketball athletes and 26 active young adults volunteered to participate in this investigation (Table 2). Participants met inclusion criteria if they were between the ages of 18 and 35 and had no recent history of lower extremity injury or surgical intervention (<12 months). NCAA athletes were in pre-season training, while the sample of young adults met inclusion if they reported engaging in moderate to vigorous physical activity for at least 3 days per week, on average. Participants were recruited on a volunteer basis and were required to provide consent via signature on an informed consent document approved by the University Institutional Review Board.

### 2.2. Procedures

Prior to completing the study protocol, participants underwent a warm-up that included a brief jog and dynamic exercises such as high knees, carioca, lateral shuffle, and jumping jacks. Participants rested for 5 min and then progressed into familiarization. In familiarization, participants were provided visual demonstration of DJ and CMJ techniques by a member of the research team. Participants were then instructed to practice both jumping techniques with monitoring and corrective feedback provided by a member of the research team, if necessary. After demonstrating proper jumping technique, participants rested for 20 min and then completed three successful DJ trials from drop heights of 0.51 m, 0.66 m, and 0.81 m, and three successful CMJ trials (12 total trials). The selection of drop heights was based on prior DJ literature. From a meta-analysis by de Villareal et al. [11], plyometric training interventions are inclusive of DJs performed from drop heights ranging between 0.12 and 1.10 m (mean ± SD = 0.49 ± 0.24 m) [11]. Further, active young adults and competitive male basketball players are observed to have similar performance on DJs from drop heights of 0.50 and 0.61 m versus drop heights ranging between 0.20 and 0.45 m, respectively [14,27,28]. Therefore, a range of drop heights between 0.51 and 0.81 m was selected with intent to elicit maximal neuromuscular reactivity from our sample of participants. The order of jumping conditions was randomized for each participant, with all three trials performed at a given condition prior to advancing to the subsequent condition. Participants rested for 1 min between trials and 5 min between conditions.

For all DJ trials, participants stood atop a custom plyometric box with dimensions of 0.51 m × 0.66 m × 0.81 m. Once atop the box, participants were instructed with the following standard verbal cue immediately prior to movement initiation: “Step forward off the box with your preferred foot, land with both feet hitting the ground simultaneously, and then immediately perform a maximal effort jump upwards as quickly and as high as possible”. Arm motion was not restricted to better represent jumps performed in real-world settings. DJ trials were monitored visually by a member of the research team and participants were asked to repeat a jump if they did not meet successful trial criterion. For instance, participants were required to land from the drop phase with both feet fully impacting an in-ground tri-axial force platform (Model FP4080, Bertec Corporation, Columbus, OH, USA). A trial was deemed unsuccessful if any portion of the feet impacted outside of the force platform.

For all CMJ trials, participants stood atop the force platform and were instructed with the following standard verbal cue immediately prior to movement initiation: “Jump upwards as quickly and as high as possible”. Arm motion was not restricted to maximize the ecological validity of results.

### 2.3. Data Analysis

#### 2.3.1. DJ

Reflective markers were affixed to participants at the location of body segment endpoints as specified by de Leva [29]. Video data were captured using a high-speed camera (300 Hz; Model EX-F1, Casio, Shibuya, Tokyo, Japan) aligned perpendicular to the sagittal plane of motion and placed at a distance of 5 m to the right of participants. The camera was levelled and secured at a height of 0.67 m above the laboratory floor. Vertical ground reaction force (GRF) data were captured from the force platform at a sampling rate of 1000 Hz. Acquisition of GRF data was initiated and terminated manually to ensure that each trial was captured in full.

Time-series data for the vertical position of de Leva [29] body segment endpoints were estimated from digitized video recordings (Kinovea, version 0.8.27). Digitization began approximately 1 s prior to movement initiation and ended at the start of the DJ flight phase. For all segment endpoints, time-series position data were passed through a low-pass, recursive, 4th order Butterworth filter set to a cut-off frequency of 6 Hz. The 6 Hz cut-off frequency was determined from residual analysis [30] and has been used previously in the literature [31]. In a custom spreadsheet (Microsoft Excel 2016, Microsoft Corporation, Redmond, WA, USA), time-series position data for the center of mass (CoM) of each body segment and the whole-body CoM were estimated using weighting tables provided by de Leva [29]. Time-series velocity data for the whole-body CoM were estimated from first central difference derivation of whole-body CoM position data. Landing impact velocity (v_i_) was estimated as the maximal downward velocity value from whole-body CoM velocity data. When performing the DJ, it cannot be assumed that drop height is equivalent to box height [32,33]. Estimates of v_i_ from video data can be used to provide insight into true drop height. In the present investigation, estimated drop height was between 14 and 16% lower than box height across DJ conditions (Table 3). The tendency for estimated drop height to be slightly lower than theoretical drop height is observed in our prior work [32] and by Baca [33].

GRF data were passed through a low-pass, recursive, 4th order Butterworth filter set to a cut-off frequency of 300 Hz. GRF data were trimmed to begin at landing impact following the drop phase and to end at the start of the depth jump flight phase. The timing of drop landing impact and depth jump take-off were determined using a RFD method [33]. Landing impact was defined as the time point when the GRF signal changed at a rate equal to, or exceeding, 10,000 N/s (e.g., 10 N ∆ between data points). Depth jump take-off was defined as the time point when the GRF signal changed at a rate below 10,000 Ns.

RSI scores were estimated using a mixed-methods approach as described by Baca [33] and Louder et al. [32]. Depth jump take-off velocity (v_t-off_; Equation (1)) was estimated by taking the difference between numerically integrated (trapezoidal rule) GRF data (v_GRF__;_ Equation (2)) and absolute v_i_. JH (Equation (3)) was estimated by inputting v_t-off_ into an equation of constant acceleration. GCT was specified as the duration of trimmed GRF data. The RSI (Equation (4)) was estimated by taking the ratio of JH to GCT. For each DJ condition, the trial corresponding with the greatest RSI score was selected for statistical analysis.

(1) $$v_{t-\mathrm{off}}=v_{\mathrm{GRF}}-\left|v_{i}\right|$$

(2) $$v_{\mathrm{GRF}}=\frac{\int^{\;}\mathrm{GRF}-\mathrm{BW}}{\mathrm{Body}\;\mathrm{Mass}}$$

(3) $$\mathrm{JH}=\left(\frac{v_{t-\mathrm{off}}{}^{2}}{19.62}\right)$$

(4) $$\mathrm{RSI}=\frac{\mathrm{JH}}{\mathrm{GCT}}$$

#### 2.3.2. CMJ

Similar to the DJ analysis, GRF data were passed through a low-pass, recursive, 4th order Butterworth filter set to a cut-off frequency of 300 Hz. CMJ take-off velocity (v_t-off_; Equation (5)) was estimated through numerical integration (trapezoidal rule) of GRF data. JH was estimated by inputting v_t-off_ into Equation (3). TTT was defined as the duration between the initiation of countermovement and CMJ take-off. Countermovement initiation was defined from visual inspection for the first 10 N decrease in the GRF signal occurring without inflection [34]. The timing of CMJ take-off was estimated using the RFD method described above [32]. RSI-mod scores (Equation (6)) were estimated by taking the ratio of JH to TTT. The CMJ trial corresponding with the greatest RSI-mod score was selected for statistical analysis.

(5) $$v_{t-\mathrm{off}}=\frac{\int^{\;}\mathrm{GRF}}{\mathrm{Body}\;\mathrm{Mass}}$$

(6) $$\mathrm{RSI}-\mathrm{mod}=\frac{\mathrm{JH}}{\mathrm{TTT}}$$

### 2.4. Statistical Analysis

The absolute and relative within-subjects reliability of RSI and RSI-mod scores were estimated using coefficients of variation (CV% = (SD × mean^−1^) × 100) and intraclass correlation coefficients (ICC), respectively. The between-subjects variability of RSI and RSI-mod scores was estimated from CV. Separate one-way repeated measures analyses of variance (RMANOVA) were performed to evaluate for main effects of jump condition [0.51 m DJ × 0.66 m DJ × 0.81 m DJ × CMJ] on RSI/RSI-mod scores, GCT/TTT, and JH. Post hoc analysis of significant main effects was performed using paired t-tests and a Bonferroni correction to the alpha type I error threshold. Effect sizes were determined using the partial eta squared (η_p_^2^) statistic. Linear regression was performed between RSI-mod (predictor) and RSI (response) scores, with Pearson correlation coefficients and coefficients of determination (*R*^2^) computed to evaluate the strength of linear association. Pearson correlation coefficients were interpreted using a scale from Campbell and Swinscow [35], which defines coefficients as very weak (*r* = 0.00–0.19), weak (*r* = 0.20–0.39), moderate (*r* = 0.40–0.59), strong (*r* = 0.60–0.79), and very strong (*r* = 0.80–1.00). Bland–Altman analysis involved estimating the measurement bias and limits of agreement (95% CI) between RSI and RSI-mod scores. Linear regression was performed on Bland–Altman data to evaluate for performance-dependent effects on measurement bias. Statistical analyses were performed in RStudio (Version 1.1.456). An alpha type I error threshold of *p* < 0.05 was used to determine statistical significance.

## 3. Results

Shapiro–Wilk tests of normality confirmed that all dependent measures were normally distributed (*p* > 0.05). The within-subjects reliability of RSI and RSI-mod scores were acceptable and similar to values reported previously in the literature (Table 4) [28,36,37]. Central tendency and dispersion results for the RSI, RSI-mod, GCT, TTT, and JH are presented in Table 5.

### 3.1. RMANOVA

There was a significant main effect of jump condition on RSI/RSI-mod scores (*F* = 22.3, *p* < 0.001) and GCT/TTT (*F* = 102.5, *p* < 0.001). Post hoc analyses revealed that RSI scores were significantly greater than RSI-mod scores (*p* < 0.001; Table 5) and that GCTs were significantly shorter than TTT (*p* < 0.001; Table 5). There was no main effect of jump condition on JH (*F* = 1.1, *p* = 0.362).

### 3.2. Linear Regression

**Figure 2:**
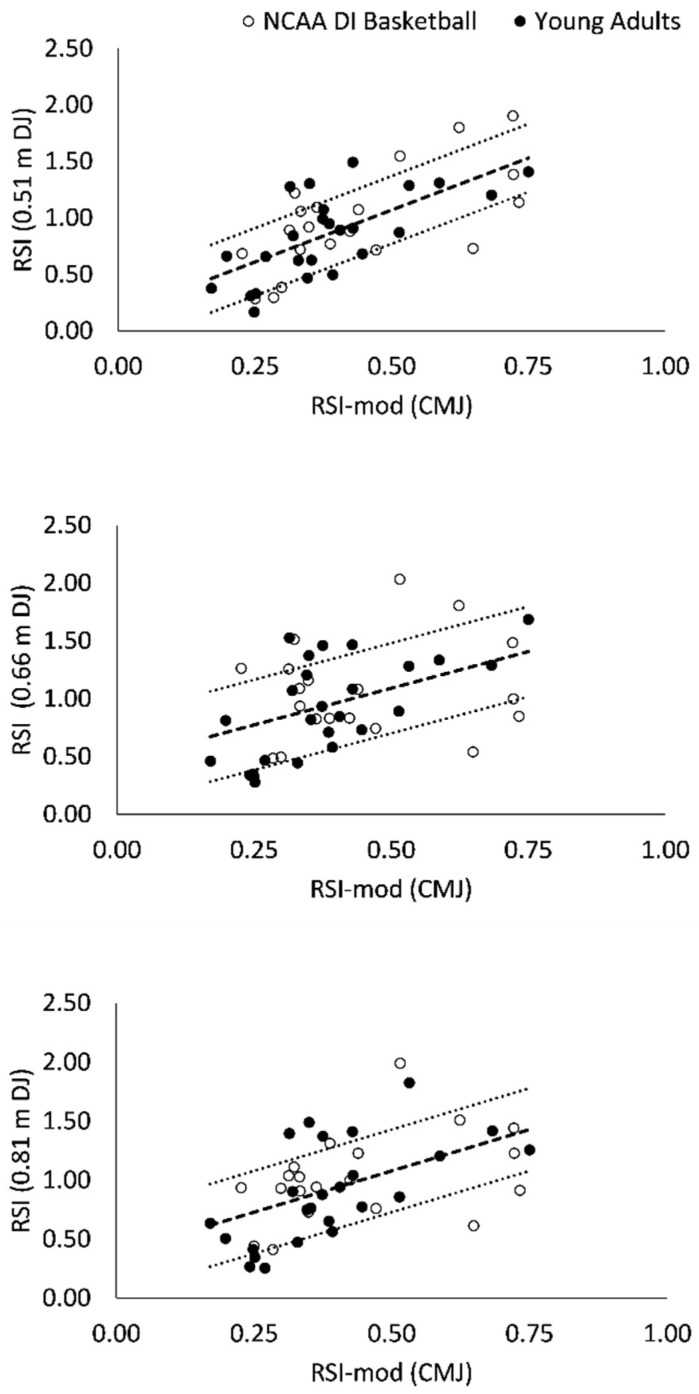
Scatter plots of reactive strength index (RSI) and reactive strength index-modified (RSI-mod) scores. Broken trendlines represent the linear regression fit ± standard error of the estimate.

Linear regression revealed significant positive associations between RSI and RSI-mod scores (*F* = 13.4–38.8; *p* < 0.001; Table 6; Figure 2). The strength of correlation between RSI-mod and RSI scores was moderate (0.66 and 0.81 m DJ; Table 6) to strong (0.51 m DJ; Table 6). The RSI-mod explained between 20 and 47% of the variance in RSI scores (Table 6).

### 3.3. Bland–Altman Agreement

**Figure 3:**
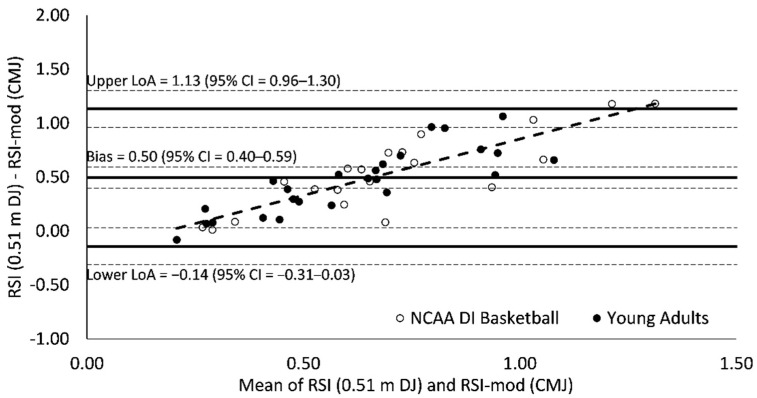
Bland–Altman plot of agreement between reactive strength index (RSI) and reactive strength index-modified (RSI-mod) scores. The broken regression line represents a significant performance-dependent effect on measurement bias (*F* = 108.4 (*p* < 0.001); *β* = 1.04 (*p* < 0.001); Intercept = −0.19 (*p* = 0.010); *r* = 0.84; *R*^2^ = 0.71; SEE = 0.18).

**Figure 4:**
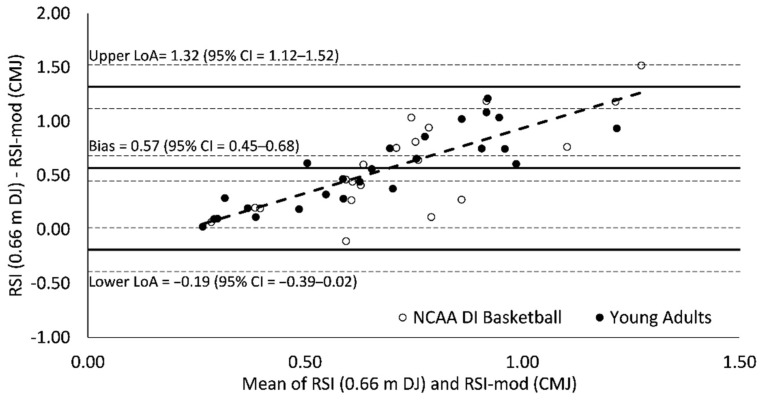
Bland–Altman plot of agreement between reactive strength index (RSI) and reactive strength index-modified (RSI-mod) scores. The broken regression line represents a significant performance-dependent effect on measurement bias (*F* = 81.0 (*p* < 0.001); *β* = 1.21 (*p* < 0.001); Intercept = −0.27 (*p* = 0.010); *r* = 0.81; *R*^2^ = 0.65; SEE = 0.23).

**Figure 5:**
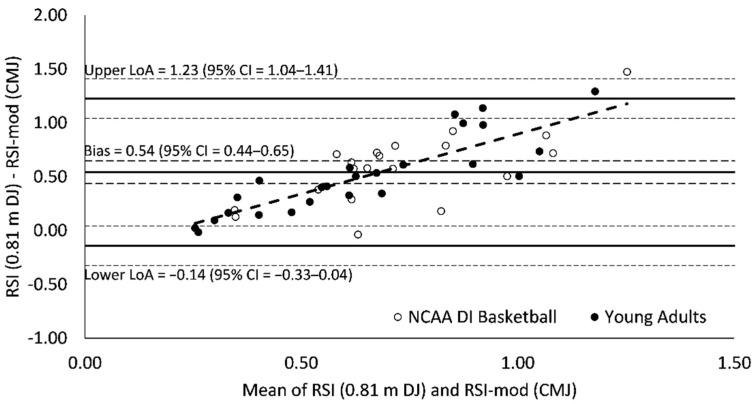
Bland-Altman plot of agreement between reactive strength index (RSI) and reactive strength index-modified (RSI-mod) scores. The broken regression line represents a significant performance-dependent effect on measurement bias (*F* = 77.2 (*p* < 0.001); *β* = 1.11 (*p* < 0.001); Intercept = −0.22 (*p* = 0.024); *r* = 0.79; *R*^2^ = 0.63; SEE =0.21).

Measurement bias (0.50–0.57) for all Bland–Altman plots was significant (95% CI = 0.40–0.68) and indicated a tendency for RSI scores to be greater than RSI-mod scores (Figure 3, Figure 4 and Figure 5). Limits of agreement between RSI and RSI-mod scores ranged from 1.27 to 1.51 (Figure 3, Figure 4 and Figure 5). For all Bland–Altman plots, linear regression revealed a significant performance-dependent effect on measurement bias, wherein the difference between RSI and RSI-mod scores was positively associated with the mean of RSI and RSI-mod scores (*F* = 77.2–108.4; *p* < 0.001; *R*^2^ = 0.63–0.71; Figure 3, Figure 4 and Figure 5).

## 4. Discussion

Our hypothesis that the RSI and RSI-mod are associative but not interchangeable measures of reactive strength was supported by the results. RSI and RSI-mod scores in the present investigation were similar to values reported previously in the literature [16,38,39] and, across all DJ conditions, RSI scores were substantially greater (+131–150%) than RSI-mod scores, a finding that was mostly attributable to longer TTT versus GCT (+115–120%).

DJ GCTs were significantly shorter than CMJ TTTs, yet they were also above the 250 ms threshold traditionally associated with a fast SSC action [9]. For DJs, participants were instructed to maximize JH and minimize GCT. Using comparable verbal instruction, Struzik [13] observed DJ GCTs below the 250 ms threshold for fast SSC action, while several authors have observed DJ GCTs that were similar to those reported in the present investigation [16,40,41,42]. To encourage a fast SSC action in DJ, it may be necessary to provide augmented feedback during familiarization or to emphasize jumping “as quickly as possible” without reference to jump height, which is observed to facilitate both shorter DJ GCTs [43] and greater RSI-mod scores [44]. In addition, we did not require participants to report a history of plyometric training. Reduced DJ GCTs [45] are noted as a potential adaptation to plyometric training, thus limited prior exposure to performing the DJ may have contributed to the GCTs observed in the present investigation. Lastly, performing DJs from a drop height that exceeds an individuals’ reactive capacity is observed to result in prolonged GCTs [14]. GCTs were not different between DJ conditions in the present investigation, which suggests that the drop heights were within the reactive strength capacity of participants.

Linear regression revealed significant positive associations between RSI and RSI-mod scores, however, the amount of shared variance (20–47%) returned from the models was moderate. This is largely consistent with the findings from McMahon et al. [16] and suggests that the RSI and RSI-mod likely do not measure the same reactive strength characteristics. From a collective view, the shared variance between RSI-mod and RSI scores in the present investigation had a tendency to exceed the 22% reported by McMahon et al. [16]. The present investigation comprised a larger sample of participants (*n* = 47 vs. 21; [16]) that were heterogeneous with respect to sex and athletic status. The heterogeneity of our sample likely resulted in a greater range of RSI and RSI-mod scores. Further, since sample size and predictor value range are two factors that can augment the shared variance returned from linear regression [46], the heterogeneity of our sample may have contributed to the differences in association observed between the present investigation and McMahon et al. [16].

The Bland–Altman analyses provide evidence of a poor agreement between RSI and RSI-mod scores. The Bland–Altman plots were consistent when RSI-mod scores were compared against RSI scores derived from 0.51, 0.66 and 0.81 m DJ. The measurement bias between RSI and RSI-mod scores (0.50–0.57) was greater than the mean for RSI-mod scores (0.42) and was statistically significant since the 95% CIs did not cross zero (95% CI = 0.40–0.68). In Bland–Altman analysis, two measures may be significantly biased yet retain strong agreement if the limits of agreement between measures is small. This was not the case in the present investigation as the ranges between upper and lower limits of agreement (1.27–1.51) were large and considerably greater than the mean values for both RSI (0.97–1.05) and RSI-mod (0.42) scores. For all Bland–Altman plots, linear regression revealed a significant performance-dependent effect on measurement bias, wherein the difference between RSI and RSI-mod scores was positively associated with the mean of RSI and RSI-mod scores. These effects suggest that there may be limited performance transfer between scores, whereby an increase in the RSI does not necessarily result in a similar increase in the RSI-mod. Further, these effects suggest that the agreement between RSI and RSI-mod scores may not be consistent when applied across populations with varied reactive strength ability.

**Figure 6:**
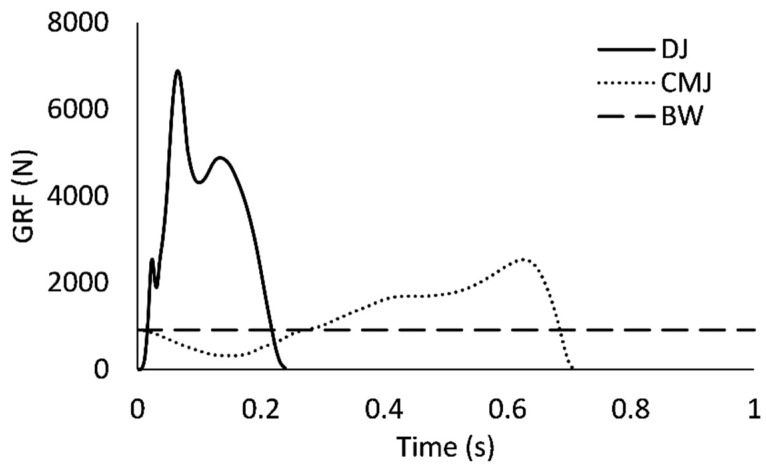
Sample vertical ground reaction Force (GRF) data for a depth jump (DJ, 0.81 m drop height) and countermovement jump (CMJ). BW = body weight.

As mentioned previously, DJ tends to elicit shorter GCT/TTT in conjunction with greater peak GRF and RFD when compared against *CMJ* (Figure 6). The DJ technique is also performed without a prolonged unweighting phase [16,26] and, as shown in Figure 6, the total duration of a fast-SSC (<250 ms) ground contact in DJ can be shorter than the duration of the CMJ unweighting phase. Differences in the GRF profiles of DJ and CMJ also infer that a greater biomechanical demand is placed on the neuromuscular system during DJ. Consequently, the RSI-mod may be the more appropriate measure of reactive strength in populations that may not have the requisite strength needed to safely and skillfully accept the high stretch-loads that are applied to the muscle-tendon complex in DJ. The RSI-mod could be used to provide partial insight into reactive strength until sufficient tolerance to the biomechanical demands of DJ is realized, at which point the RSI may then be the preferred measure.

From a neuromotor control perspective, the self-initiated drop and landing impact phase of DJ may represent the most important distinction from CMJ. In DJ, the neuromotor system must develop a planned motor response to landing impact in anticipation of the timing and magnitude of GRF [22]. This feedforward control strategy is executed during the drop phase, resulting in the pre-activation of lower extremity skeletal muscle prior to landing impact [22]. Pre-activation increases the stiffness of skeletal muscle and, when impact forces are predicted correctly, facilitates a safe dispersion of stress through the muscle-tendon complex upon landing [22]. Pre-activation may also enhance the SSC response to landing impact by preparing the muscle-tendon complex to store elastic energy and by modifying the short latency spinal reflex via input from supraspinal drive and alpha-gamma co-activation [7,22,47,48].

Several authors have acknowledged the pre-activation of skeletal muscle as a fundamental component of natural, or functional, lower-extremity SSC actions [48,49,50]. For example, to evaluate functional lower-extremity SSC utilization, Nicol et al. [49] recommend the performance of jumping techniques, such as DJ, that involve a rapid stretch of pre-activated skeletal muscle. From this perspective, DJ-derived RSI scores may be more representative of natural sport movements (e.g., running, sprinting, cutting and jumping) that evoke robust lower-extremity SSC actions in response to impact between the feet and the ground. In contrast, RSI-mod scores are derived from a controlled CMJ technique performed with the feet remaining in contact with the ground for the entire duration of the unweighting and vertical jump phases. RSI-mod scores are considered a valid and reliable assessment of slow lower-extremity SSC utilization and neuromuscular power [51,52,53,54], yet their application as a measure of functional lower-extremity reactive strength, or SSC utilization, may need to be reconsidered.

An informal search of the literature (Figure 1) reveals a recent and increasing interest in the RSI and RSI-mod. Using the search term “reactive strength index”, Google Scholar (http://scholar.google.com (accessed on 15 March 2020) returned 252 and 42 peer-reviewed manuscripts from 2000 to 2020 that feature the RSI or RSI-mod as a dependent measure, respectively (Figure 1). Notably, among the 42 manuscripts that included the RSI-mod as a dependent measure, 8 (19%) incorrectly refer to the RSI-mod as the RSI. The results from the present investigation and McMahon et al. [16] are evidence that the RSI and RSI-mod cannot be used interchangeably, yet, with the inconsistent application of the terms in the literature, it may be of practical value to consider further distinction between the measures. One example would be to revise the nomenclature of the RSI-mod to the Explosive Strength Index (ESI), which may better reflect the biomechanical and neuromotor demands of CMJ.

It is important to mention several limitations of the present investigation. First, we estimated the JH component of the RSI using a take-off velocity method. This approach required an estimation of DJ landing impact velocity taken from digitized 2-dimensional video. Video data were digitized in accordance with the de Leva [29] anthropometric model which, when compared against criterion methods, yields valid estimates of whole-body CoM displacement [55]. Estimating DJ take-off velocity through a combination of videography and force platform dynamometry is supported in the literature and may address the known threats to validity associated with estimating JH from flight time [32,33]. Regardless, there are several potential sources of measurement error that arise from the capture and digitization of 2-dimensional video, thus a recommendation for future investigation is to consider estimating DJ landing impact velocity using criterion methods, such as optical motion capture. Second, we instructed participants to jump as high and as quickly as possible. Our approach to verbal instruction likely contributed to DJ GCTs that exceeded the threshold for a fast SSC action. As such, the results of the present investigation should be considered in context with the specific verbal instructions provided to participants and DJ GCTs. Lastly, it is important to note that RSI scores were not different across DJs performed from varying drop heights. While this has been observed in prior literature [27], it also brings into question the sensitivity of the RSI as a measure of reactive strength. For instance, achieving similar RSI scores in DJs performed from a low versus high drop may not account for differences in the amount of mechanical energy absorbed during landing impact. While the RSI is reliable and valid as a broad metric of DJ performance, there may be value in focusing future research on a more specific metric of reactive strength, which may include a direct analysis of the rate of absorption and production of mechanical energy.

## 5. Conclusions

This is the first investigation to report on the association and agreement between RSI and RSI-mod scores acquired from a mixed-sex sample of collegiate athletes and active young adults. Results from linear regression and Bland–Altman analysis give evidence that the RSI and RSI-mod are associative but not interchangeable measures of reactive strength. This is not surprising when considering that DJ and CMJ are unique from biomechanical and neuromotor perspectives. The results herein inform researchers and practitioners to consider the RSI and RSI-mod as specific measures of agility that provide unique information with regard to reactive strength, or lower-extremity SSC utilization.

## Figures and Tables

**Table 1:** Reactive strength assessments.

Assessment	Computation
Reactive Strength (slow SSC; Young [8])	${\mathrm{JH}}_{\mathrm{CMJ}}-{\mathrm{JH}}_{\mathrm{SJ}}$
RSI (fast SSC; Young [8])	$\frac{\mathrm{JH}}{\mathrm{GCT}}$
RSI-mod (slow SSC; Ebben and Petushek [15])	$\frac{\mathrm{JH}}{\mathrm{TTT}}$

SSC = stretch-shortening cycle; CMJ = countermovement jump; SJ = squat jump; RSI = reactive strength index; RSI-mod = reactive strength index-modified; JH = jump height; GCT = ground contact time; TTT = time to take-off.

**Table 2:** Participant Characteristics.

	NCAA DI Basketball		Young Adults	
	Male	Female	Male	Female
*n*	10	11	13	13
Age (years)	20.1 (1.3)	19.6 (0.8)	23.9 (1.7)	23.3 (1.8)
Body mass (kg)	91.6 (11.8)	74.4 (10.3)	80.2 (12.5)	68.0 (14.5)
Height (cm)	196.9 (8.0)	181.0 (8.3)	177.5 (8.4)	167.3 (8.6)

Values are reported as mean (SD).

**Table 3:** Depth jump (DJ) landing impact velocities (v_i_) derived theoretically from box height and from 2-dimensional videography.

Condition	Theoretical v_i_ (m s^−1^)	Estimated v_i_ (m s^−1^)	Estimated Drop Height (m)
0.51 m DJ	3.16	2.94 (0.39)	0.44 (0.10)
0.66 m DJ	3.60	3.31 (0.29)	0.56 (0.09)
0.81 m DJ	3.99	3.65 (0.17)	0.68 (0.06)

Values are reported as mean (SD).

**Table 4:** Within-subjects reliability of RSI and RSI-mod scores.

Measure	RSI (0.51 m DJ)	RSI (0.66 m DJ)	RSI (0.81 m DJ)	RSI-mod (CMJ)
ICC	0.91 (0.87–0.95)	0.89 (0.84–0.94)	0.88 (0.83–0.94)	0.95 (0.93–0.97)
CV; %	12 (11–13)	13 (11–15)	15 (13–17)	8 (6–9)

DJ = depth jump; CMJ = countermovement jump; RSI = reactive strength index; RSI-mod = reactive strength index-modified; ICC = intraclass correlation coefficient; CV = coefficient of variation. Results are presented as ICC/CV (95% CI).

**Table 5:** Effects of jump condition on dependent measures.

Variable	0.51 m DJ	0.66 m DJ	0.81 m DJ	CMJ	*η_p_*^2^ (95% CI)
RSI/RSI-mod	0.97 (0.46) *	1.05 (0.49) *	1.03 (0.52) *	0.42 (0.16)	0.15 (0.06–0.23)
JH (m)	0.37 (0.15)	0.39 (0.15)	0.40 (0.16)	0.35 (0.12)	0.02 (0.00–0.04)
GCT/TTT (s)	0.41 (0.11) *	0.40 (0.11) *	0.41 (0.10) *	0.88 (0.26)	0.37 (0.27–0.47)

DJ = depth jump; CMJ = countermovement jump; RSI = reactive strength index; RSI-mod = reactive strength index-modified; JH = jump height; GCT = ground contact time; TTT = time to take-off. Data are reported as mean (SD); * Significantly different from CMJ (*p* < 0.001); *η_p_*^2^ = partial eta squared effect size.

**Table 6:** Linear regression models.

Predictor	Response	*F*	*β*	Intercept	*r*	*R* ^2^	SEE
RSI-mod	RSI (0.51 m DJ)	38.1 *	0.26 *	0.18 *	0.69	0.47	0.30
RSI-mod	RSI (0.66 m DJ)	11.0 ^Ұ^	0.16 *	0.25 *	0.45	0.20	0.39
RSI-mod	RSI (0.81 m DJ)	16.7 *	0.20 *	0.22 *	0.53	0.28	0.35

RSI-mod = reactive strength index-modified; RSI = reactive strength index; DJ = depth jump; *r* = Pearson correlation coefficient; *R*^2^ = coefficient of determination; SEE = standard error of the estimate. * *p* < 0.001. ^Ұ^ *p* = 0.001.
